# Development and Preliminary Validation of the PC-FCQ: The Parent and Carer Food Choice Questionnaire

**DOI:** 10.3390/nu17101735

**Published:** 2025-05-20

**Authors:** Alex McKenna, Rachael L. Thurecht, Libby Swanepoel, Georgia Blair, Fiona E. Pelly

**Affiliations:** School of Health, University of the Sunshine Coast, Maroochydore, QLD 4558, Australia; alex.mckenna@health.qld.gov.au (A.M.); rthurech@usc.edu.au (R.L.T.); lswanepo@usc.edu.au (L.S.); georgia@kidsatplaytherapyservices.com.au (G.B.)

**Keywords:** determinant, factor, toddler, diet, nutrition, survey

## Abstract

**Background**: Poor nutrition in childhood can have irreversible impacts on development and health, potentially resulting in cognitive impairments and an increased risk of chronic disease later in life. **Aim**: This study aimed to develop and validate the Parent Carer-Food Choice Questionnaire (PC-FCQ) to determine the key factors influencing the parental food choices of children aged between two and five years. **Methods**: A sample of 737 Australian parents and carers completed the questionnaire consisting of 98 items on a 10-point frequency of influence anchored at 1 never to 10 always. Principal component analysis (PCA) was employed to identify the key factors and refine the questionnaire. **Results**: The PCA extracted 65 items organized into 12 factors explaining 62.8% of the total variance. The factors were named *ethical concerns*, *nutritional attributes*, *child preferences*, *child mood*, *awareness*, *parent sensory*, *convenience*, *situation*, *culture*, *professional advice*, *accessibility* and *weight control*. The final Kaiser–Meyer–Olkin measure was 0.93, and the Bartlett test of sphericity was statistically significant X2(4753) = 39,643.87, *p* < 0.001. Moderate intercorrelations were detected between seven factors. **Conclusions**: This research resulted in a PC-FCQ that includes factors specifically relevant to parental food choice. The PC-FCQ will enable researchers and clinicians to more effectively provide nutrition education and dietary interventions to suit the parent and child. The next phase of this research will be to test the accuracy and reliability of the PC-FCQ in an independent sample via confirmatory factor analysis.

## 1. Introduction

Poor nutrition in childhood can have irreversible impacts on development and health, potentially resulting in cognitive impairments and an increased risk of chronic disease later in life [1,2]. Eating patterns in childhood are predictive of those in adulthood, with dietary habits becoming increasingly difficult to change with age [1,2,3]. Furthermore, according to the World Health Organization (WHO), Improving early childhood development: WHO guideline [4], poor dietary intake can impact educational attainment and economic productivity, and have intergenerational ramifications. To support the development of healthy eating habits from an early stage, there has been mounting interest in understanding the determinants of food choice for parents and carers of young children. This is particularly relevant to very young children prior to commencing school who are being exposed to food and meal variety for the first time predominately by their parents or carers [5,6,7].

Food choice is complex and influenced by multiple physiological, psychological, social, and economic factors and is continuously evolving due to globalisation, urbanisation, and migration, alongside ever-shifting traditions and lifestyle [8,9,10]. The multifaceted nature of food choice in young children is complicated by their reliance on their parents and carers [5,7]. Parents play a crucial role in childhood exposure to food and studies have demonstrated that parental dietary intake can influence children’s food intake through modelled behaviour [11,12]. Parental role modelling is one of over 300 determinants relevant to children’s food choice mapped in the Determinants of Nutrition and Eating (DONE) framework [13].

There is a growing body of research investigating food choice in different populations and the impact on dietary patterns [6,14]. The Food Choice Questionnaire (FCQ), developed in 1995, is one of the most extensively used tools to examine food choice [15]. This was developed using exploratory factor analysis to extract nine latent constructs (factors) and 36 items, by principal component analysis (PCA), then psychometric analysis utilising confirmatory factor analysis and test–retest reliability to establish the validity of the tool [15]. A number of modified versions of the FCQ have been proposed to include contemporary factors of environmental considerations, political values, and religion [16,17,18,19]. Principal component analysis is considered a suitable approach in food choice questionnaire development and validation through the use of statistical analysis to extract meaningful factors for a more concise questionnaire [20].

Food choice motives specific to parents of young children aged 2 to 5 years have been explored using a modified, but unvalidated, version of the FCQ that included an addition of six items relating to parental food choice with altered wording to suit the child [6]. Using this tool, results demonstrated that parents were highly motivated by health and nutrition; however, this did not translate into the food preferences of their young children [6]. Various studies have also explored individual determinants of food choice, such as family conditions, allergies and intolerances, convenience, and mood, as well as broader sociological impacts such as cost, marketing, and availability predominately using qualitative methodology [21,22,23,24,25]. The in-depth exploration of parent perspectives using these methods can inform the development of questionnaires that can allow the exploration of multiple factors concurrently, subsequently allowing for the ranking of factors in order of their influence. To the best of our knowledge, a validated tool that assesses multifaceted determinants of parental food choice for young children has not been developed. Therefore, the aim of this study was to develop and validate the Parent-Carer Food Choice Questionnaire (PC-FCQ), a questionnaire that investigates the determinants of parent and carer food choice for young children. This is the first step in establishing the validity and reliability of a behavioural questionnaire [26].

## 2. Materials and Methods

### 2.1. Preliminary Questionnaire Development

This validation study included the development of a preliminary questionnaire with a pool of question items on the determinants of food choice and a data-driven statistical procedure to develop the PC-FCQ. The items in the draft questionnaire were informed by structure of Steptoe’s FCQ [15], a scoping review on food choice influences for parents and young children conducted by the authors (unpublished) [27] and the DONE framework [13,14]. The research team and two end users reviewed and revised the questions to ensure clarity and readability. The final questionnaire contained 98 items possibly influencing parent-carer and child food choices. The 98 items broadly represented twenty theoretical groupings underpinned by the existing literature (named as; health and nutrition, emotion of child, emotion of parent, convenience, sensory appeal, natural content, price, weight control, familiarity to child, ethical and environmental welfare, animal welfare, political and social justice values, religion and cultural considerations, familiarity to parent, marketing and brand, influence of others, perceived quality, freshness, variety, satiety, food literacy, and situational factors). Each item was presented as a neutral statement in a randomised order, and participants were asked to answer the item in relation to how much it influenced their food choices on a 10-point frequency scale anchored at 1 never to 10 always. A 10-point frequency scale was employed as it is demonstrated to be easy to use and preferred by participants, whilst still providing reliable and valid data [28]. The questionnaire was reviewed by the research team and two independent researchers with expertise in this field, and feedback was provided on the language and relevance of questionnaire items. Content validity was determined by examining the items against elements of the DONE framework [13], the original FCQ [15], and the current literature on determinants of food choice in the general population as well as parents and children [6,16,19,29,30]. In addition, researchers considered empirical translation and theoretical and practical concerns in the questionnaire design. Socio-demographic descriptions were collected including gender, children characteristics, marital status, education level, employment status, and postcode as well as questions about nutrition knowledge and family dynamic. The questionnaire was administered online via Qualtrics where participants completed it anonymously.

### 2.2. Participants

A convenience sample of parents and carers was invited to complete the online questionnaire between May and August 2023. This study was promoted via social media and organisations such as day-care centres, schools, and playgroups. These organisations were contacted nationwide to seek responses spanning a wide range of regions and demographics. A target sample of 980 was set to achieve the recommended 10:1 participant-to-item ratio for PCA [20]. To entice participation, an incentive prize for participants having a chance of winning one of three meal-kit service boxes was utilised.

This study was conducted in accordance with the Declaration of Helsinki, and the protocol was approved by the Human Research Ethics Committee of the University of the Sunshine Coast (S221776) on 7 December 2022. Informed consent was obtained from all individual participants included in this study.

### 2.3. Data Analysis

Data were exported to Microsoft Excel (version 16) for cleaning, and the Statistical Package for Social Sciences Statistical Software (SPSS, version 29) was used for data analysis. Sampling adequacy needed to be assessed through items receiving at least one correlation > 0.3, a Kaiser–Meyer–Olkin (KMO) measure >0.8, and a significant Bartlett’s test of sphericity [20,31,32]. All iterations applied an oblique Promax rotation that assumed correlation between factors [20]. Cronbach’s alpha (C-α) was utilised to analyse the internal reliability of the extracted factors; a value of >0.6 denotes acceptable values [33]. Composite scores were computed for each factor based on the mean response across the indicator items for that factor. The normality of distribution of each factor was assessed via the Shapiro–Wilk score [34]. Spearman’s rank order correlation was employed to examine the inter-factor correlation. The level of significant for the study was *p* < 0.05. The socio-economic status was determined via The Index of Relative Socio-economic Advantage and Disadvantage [35].

## 3. Results

The questionnaire had a full sample response rate of 1137, and the sample used for analysis was 737. Participants removed included those that did not live in Australia (*n* = 8), who were not a primary carer of a child aged between two and five years old (*n* = 15), did not consent to participating in the survey (*n* = 3), and those with unanswered items relevant to food choice (*n* = 374). The demographic characteristics are described in Table 1. The participants had a median age of 35 years (range 21–67 years), with the majority identifying as female. The sample included, but was not limited to, biological parents, stepparents, grandparents, foster parents, and guardians.

### 3.1. Principal Component Analysis

The sample had a case-to-item ratio of 7.3:1, exceeding the minimum 5:1 ratio considered for factor analysis [20]. The initial analysis demonstrated sampling adequacy and that the dataset was appropriate for factor analysis as indicated by all items receiving at least one correlation >0.3, an overall KMO measure of 0.93, and a significant Bartlett’s test of sphericity X2(4753) = 39,643.87, *p* < 0.001 [20,31,32]. A latent factor is best represented by a higher loading item, with <0.4 regarded as minimally acceptable [20]. Multiple PCA iterations were conducted while removing items each time until a simplified model of items into component factors could be extracted. Multiple PCA iterations were conducted. In total, 33 items were removed for low loading <0.4 or for loading across more than one factor (cross-loading). The final rotation contained 65 items across twelve factors with eigenvalues between 14.74 and 1.14, explaining 62.80% of the total variance (*n* = 737, after listwise deletion). The KMO was 0.92 and the Bartlett’s test of sphericity was significant (X2(2080) = 26,845.73, *p* < 0.001). All communalities were >0.45.

The labels for the factors were derived from the highest loading item, which theoretically explained the most variance, in consideration with other loading items, the existing literature on the FCQ, and consultation with the research team [20]. The twelve factors were labelled *ethical concerns*, *nutritional attributes*, *child preferences*, *child mood*, *awareness*, *parent sensory*, *situation*, *convenience*, *culture*, *professional advice*, *accessibility*, and *weight control*. Using Cronbach’s alpha (α), the internal reliability was tested, as demonstrated in Table 2. All factors had adequate scoring for exploratory research (>0.6), and eleven factors exceed (>0.7) the more generalised lower limit [20]. None of the Cronbach’s alpha scores could be improved with the removal of any items and as such, all items were retained.

### 3.2. Intercorrelations Between Factors

The distribution of the factor scores was non-parametric (w > 0.85, *df* 735, *p* < 0.001) and Spearman’s rank-order analysis was applied to assess intercorrelations between factors (Table 3). Most of the significant correlations noted were considered weak. There were seven moderate correlations (rs 0.40 to 0.59) [20]. *Weight control* was moderately correlated to *ethical concerns* (rs (737) = 0.417 *p* < 0.001) and *professional advice* (rs (737) = 0.415, *p* < 0.001)*. Nutritional attributes* was moderately correlated with *ethical concerns* (rs (737) = 0.532 *p* < 0.001) and *weight control* (rs (737) = 0.494 *p* < 0.001). There was a moderate correlation between *awareness* and *child mood* (rs (737) = 0.502 *p* < 0.001) and *professional advice* (rs (737) = 0.466 *p* < 0.001). Finally, *child preferences* was moderately correlated with *child mood* (rs (737) = 0.588 *p* < 0.001).

The final PC-FCQ has been provided as Supplementary Material (S1: PC-FCQ: The Parent and Carer Food Choice Questionnaire).

## 4. Discussion

The primary aim of this study was to develop and validate the PC-FCQ with a sample of parents and carers of children aged between two and five years. This is the first study to develop a tool specific to this population following an evidenced-based and data-driven process for identifying latent constructs. Through exploratory factor analysis, PCA was completed to identify 12 factors, including 7 similar to the FCQ (*ethical concerns*, *nutritional attributes*, *child preferences*, *child mood*, *parent sensory*, *convenience*, and *weight control*), and 5 new (*awareness*, *situation*, *culture*, *professional advice*, *accessibility*) and 65 items. This analysis explained 62.8% of the total variance which is deemed adequate within social sciences; additionally, each factor met the minimally acceptable threshold for internal reliability [20]. The results of this study were similar to the original FCQ, which identified nine factors accounting for 65.2% of the total variance [15]. 

The factors extracted in the PC-FCQ align with themes within the existing literature regarding food choice, and the items have grouped in a theoretically sound way. The PC-FCQ and the original and modified versions of the FCQ demonstrate parallels in multiple of the extracted factors; however, slight changes to some factor names were necessary in the PC-FCQ to ensure contextual specificities for this population were represented. Specifically, the *nutritional attributes* factor in this study aligns with *health* and *natural content*, *Child mood* aligns with *mood*, and *parent sensory* aligns with *sensory appeal*, respectively [15]. Factors emerged as new constructs that were specifically relevant to either parents or the influence of the child (*child preferences*, *child mood*, *parent sensory).* The factor *weight control*, while similar to the FCQ, included items relevant to both child and adult weight. This has been observed in similar cross-sectional studies investigating parental food choice in comparable settings [6]. Due to the PCA method used and the extensive item pool, new factors emerged that were not previously reflected in the original [15], and were labelled *accessibility*, *awareness*, *situation*, *culture,* and *professional advice*. The factor *accessibility* covered the cost of food (labelled as *price* in FCQ) as well as the location and availability of food.

The factor named *awareness* encompasses items relating to marketing and credibility. Awareness is a critical component in shaping consumer behaviour, as it involves the recognition and recall of products and brands clearly reflected in this factor with ‘media advertisement’ and ‘product endorsement on label’ being prominent strategies used to increase awareness among consumers. Awareness and credibility are closely linked and significantly affect brand consideration and choice for consumers [36]. The inclusion of items like ‘promotion through organisations’ and ‘product endorsement on label’ reflects the importance of trusted sources and endorsements in raising awareness. These promotional activities, often conducted by reputable organizations such as schools and health clinics, can enhance the perceived credibility and familiarity of food products.

Familiarity with a brand or product, as indicated by the items ‘trust in the brand/product’ and ‘familiar to me’, also plays a significant role in awareness. Consumers are more likely to choose products they recognize and trust, which can lead to increased preference and loyalty. Additionally, Russell et al. (2017) found that front-of-pack marketing attributes significantly influence parental food choices, underscoring the power of familiarity and awareness in food selection [37]. The construct of awareness in the PC-FCQ thus captures the interplay between marketing efforts that raise awareness and the familiarity that builds trust. This dual influence is essential for understanding how parents and carers make food choices for their children. By leveraging both awareness and familiarity, food marketers can effectively influence consumer behaviour, leading to better-informed and more confident food choices.

A factor labelled *situation* emerged in the PC-FCQ, a dimension that was not reflected in the original FCQ. This factor includes the items of ‘time of day’, ‘time of meal’, and ‘activities of the day’ that reflect the temporal influences that can impact food choice, considering aspects like activity, context, and routine [29,38,39]. Similar themes were explored in a 2014 paper that identified situational factors as influential in food choice which used a modified version of the original FCQ [29]. This aligns with the findings that decisions about time allocation are individual and subjective, encompassing both social and cultural factors [40]. This is compounded in a family setting when considering the structure and stability that is often provided by a routine [39]. There is an established relationship between eating-related family routines and the nutritional outcomes of children, highlighting the importance of understanding the situational aspects of the day [39]. For parents of young children, this factor could represent time-scarcity, a recognised factor for opting for convenient foods, often when away from the home [40]. Another new factor named *culture* with items related to religion, as well as cultural and social norms, was identified in the PC-FCQ. This finding is unsurprising and consistent with the more recent literature on food choice, which captures the significance of various cultures in conserving traditional meals and food items [41,42]. A component of this factor relevant to religion was identified in the 2000 modified version of the FCQ [19]. Thus, it reflects the social context of parents, which may influence their comfort level when introducing different food items to their child [41,42].

While the factor *ethical concern* has been explored in both the original FCQ [15] and modified versions [16,19], this study identified eleven items with high internal reliability [17,18,19,43]. This factor accounts for the most variance within the analysis, in contrast to the original FCQ which the ethical factor accounts for the least amount of variance [15] and has been viewed as problematic in the literature [18,19]. Given the more current nature of the PC-FCQ, it is unsurprising that there is a heightened emphasis on sustainability and ethical food choices. Therefore, these findings may reflect the increased public awareness and concern surrounding these topical issues [44]. The factors within the PC-FCQ may represent a more contemporary and pertinent tool, aligning with the growing emphasis on sustainable diets and planetary health.

A new factor labelled *professional advice* emerged in the PC-FCQ. This factor comprises guidance from dietitians or nutritional professionals, doctors or other medical professionals, and exercise or fitness professionals. The research focused on childhood obesity, weight management, and the guidance provided by paediatric health professionals indicates that health professionals are a key source of food and nutrition advice for parents [45,46,47]. It is important to note that the high socioeconomic status of the sample in our study may have impacted the findings for this factor. Often, parents of higher socio-economic backgrounds are more likely to have increased health literacy which may be a protective factor in accessing health services and maintaining good health [48,49]. This is compounded by parents of lower socio-economic status often being less likely to engage with health services; despite often having increased rates of injury and illness [48,49]. As this factor may be relevant to those who participated in this study, confirmatory factor analysis with a different sample is recommended for further testing of the PC-FCQ’s construct validity and reliability.

There were various significant intercorrelations identified within the final iteration of extracted factors. Intercorrelations were to be expected as an oblique rotation was employed which assumes factors are linked [20]. Moderate positive correlations were identified within seven factors; however, none were high enough (>0.07) to indicate multicollinearity [20], which is an issue which supports the validity of our novel tool. Among the PC-FCQ factors, the strongest correlation was between *child mood* and *child preferences.* Positive moods often have more favourable effects on food choice, whereas negative moods often lead to energy-dense and nutrient-poor choices [50]. There was also a moderate correlation between *weight control* and *professional advice* which is likely due to the management of weight control by paediatric health professionals [45,46,47]. Unsurprisingly, *weight control* was also moderately correlated with *nutritional attributes.* This is likely due to the control that parents have over food choice for their children, which encompasses the many aspects explored in the *nutritional attributes* factor including but not limited to ‘Effect on my child’s health’, ‘Fat content’, ‘Protein content’, ‘Quality and freshness’, and ‘Vitamin and mineral content’. This may also reflect the higher socio-economic and education status of participants, particularly in seeking professional advice. Finally, there was a moderate correlation between *awareness* and *child mood*. This aligns with the findings that suggest repeated product exposure targeted at children can build preference, which can in turn influence parental food choice [37,51].

We note that questionnaires developed in certain contexts may lack validity in others, lending itself to the importance of testing reliability of the PC-FCQ. A Finnish study used a modified version of the FCQ to assess parental food choice motives and extracted eight factors, similar to the FCQ [5]. It is important to note that this questionnaire only included 27 of the original 36 items from the FCQ which may explain the extraction of less factors. Similarly, The Child Feeding Questionnaire was developed in 2001 and included a PCA and confirmatory factor analysis to validate the tool [52]. However, this questionnaire was primarily focused on parental control and included factors relating to parental beliefs regarding obesity proneness and parental control practices and attitudes concerning child feeding [52,53].

Several individual items were not included in the PC-FCQ as they did not align with a factor, or they cross-loaded (indicative that the item is not measuring a single factor). Items not aligning with a factor, for example, items relating to food allergies and intolerances and medical conditions, were excluded from the PC-FCQ. An investigation into whether they should be included as single items is warranted as there have been many studies that have explored the impact of such influences on food choice [21,22,23,24,25]. For example, heightened vigilance from parents and carers may be present when children have food allergies, which can result in unhelpful coping strategies and avoidance of uncertain food environments [54,55]. In addition to this, feeding problems and food selectivity are common in children with developmental disabilities which can influence parental food choice [56,57].

There are several strengths in the design of this study. The PC-FCQ incorporated simple and neutral phrasing for items to ensure the same interpretation across the sample Additionally, a 10-point frequency scale was applied instead of a 4-item Likert scale which was identified as a limitation of the original FCQ [18] and further adapted versions have employed either a 5- or 7-point scale to mitigate this [58,59,60]. This heightens the sensitivity of the data collected to increase the variability as well as the addition of a neutral point to avoid a forced preference on participants [18,61]. The greater variance can also result in less skewed data and is comparable to 5- and 7-point scales for exploratory factor analysis.

There are several limitations with this study that need to be acknowledged. The sample was not demographically representative of the broader population, with 96.8% of participants identifying as female and 63.5% holding tertiary qualifications. Selection bias of those available and have an interest in nutrition are potentially more likely to have participated. This overrepresentation may limit the generalizability of the findings. However, given that primary carers of children aged two and five years is more commonly undertaken by women [62], the sample may still reflect the population of focus and in turn enhance internal validity of findings. Although representative samples across different demographic groups are warranted to ensure the results of the study are generalisable to the broader Australian population [63]. Additionally, the survey had a high rate of incomplete responses (*n* = 374) which may be due to the length of the questionnaire and the often time-poor nature of parents with young children. Although this decreased the potential case-to-item ratio, the 7.3:1 ratio achieved is greater than the minimum (5:1) recommended for minimizing risk of deriving sample-specific factors giving greater confidence in the generalisability of the PC-FCQ [20]. There is also potential for social desirability bias despite responses being anonymous. Therefore, we recommend that the factorial structure of the PC-FCQ is tested in an independent sample to examine reproducibility, construct validity, and generalisability. Furthermore, this study was cross-sectional in nature and does not consider the stability of the PC-FCQ over time.

## 5. Conclusions

The newly developed and validated PC-FCQ, consisting of twelve latent factors and 65 items, is a valuable addition to researchers and clinicians working within the area of childhood nutrition and eating behaviours. The uniqueness of the PC-FCQ is the inclusion of items specific to both parents and children with the factors *child mood*, *parent sensory*, *awareness*, and *child preferences*, in addition to the factors alike those in the original FCQ. Additional new factors of *culture*, *situation*, *awareness*, and *professional advice* were congruent with the current literature on the determinants of food choice and influences of eating behaviour in this population. Future directions will be to establish construct validity through confirmatory factor analysis by testing this questionnaire within an independent sample, and to consider the inclusion of single items such as food allergies and intolerances and medical conditions. This will aid in guaranteeing the robustness of the questionnaire, making it suitable for a wider application within parents and carers of children aged between two and five years.

## Figures and Tables

**Table 1:** Demographic characteristics of the sample of parents and carers (*n* = 737) of children aged between 2 and 5 years in Australia.

		*n*	%
Parent gender identity	Male	19	2.6
	Female	713	96.7
	Unspecified/Other ^a^	5	0.7
No. of children (2–5 years)	1	571	77.5
	2	160	21.7
	3	6	0.8
Marital status	Single	48	6.5
	Married	508	68.9
	Separated/divorced/widowed	25	3.4
	De-facto	148	20.1
	Other	8	1.1
Highest level of education completed	Lower secondary school	12	1.6
	Upper secondary school	65	8.8
	Vocational education and training	192	26.1
	Undergraduate or equivalent	285	38.7
	Postgraduate or equivalent	183	24.8
Employment status	Full time employment	192	26.1
	Part time/casual employment	356	48.3
	Self employed	32	4.3
	Unemployed	108	14.7
	Stay at home parent	10	1.4
	Other	39	5.3
SEIFA Status ^b^	1—Greatest disadvantage and lack of advantage	101	13.7
	2	111	15.1
	3	178	24.2
	4	188	25.5
	5—Lack of disadvantage and greater advantage	159	21.6

^a^ Includes non-binary and unspecified responses. ^b^ Socio-economic Indexes for Areas (SEIFAs) based on postcode in relation to The Index of Relative Socio-economic Advantage and Disadvantage.

**Table 2:** Summary statistics from the exploratory factor analysis of the Parent-Carer Food Choice Questionnaire; factor loadings; communalities; internal consistency (α); and variance explained (%).

Factor and Items	Factor Loading	Communalities
*Ethical concerns* (α = 0.939, 22.67%)		
Pain inflicted on animals	0.874	0.672
Respecting animal rights	0.871	0.733
Being animal friendly	0.876	0.742
Freedom of movement of animals	0.844	0.670
The impact on the environment	0.795	0.724
Environmentally friendly production/manufacturing	0.753	0.740
CO_2_ emissions from production	0.748	0.667
Free range production	0.739	0.628
Environmentally friendly packaging	0.722	0.636
Disruption to the ecosystem	0.638	0.636
Produced in a country where human rights are not violated	0.630	0.565
*Nutritional attributes* (α = 0.895, 10.61%)		
Vitamin and mineral content	0.829	0.666
The amount of natural ingredients	0.812	0.685
The amount of artificial ingredients	0.747	0.636
Quality and freshness	0.702	0.588
Protein content	0.700	0.606
Effect on my child’s health	0.697	0.544
Fibre (roughage) content	0.638	0.580
The amount of additives	0.629	0.541
Providing variety to my child/children	0.590	0.473
Fat content	0.546	0.581
*Child preferences* (α = 0.878, 5.54%)		
My child’s taste preferences	0.857	0.743
Acceptance by my child	0.831	0.618
Familiar to my child/children	0.719	0.630
Enjoyment by my child	0.701	0.608
My child’s texture preference	0.697	0.593
Requested by my child	0.682	0.541
Previous food my child has eaten	0.650	0.528
Satisfies my child (fills them up)	0.415	0.517
*Child mood* (α = 0.886, 4.12%)		
Helping my child relax	0.910	0.747
Keeping my child alert	0.821	0.687
Soothing my child	0.790	0.664
How tired they are	0.769	0.609
Helping to pacify my child	0.729	0.653
Rewarding my child	0.543	0.531
Easy for my child to eat	0.453	0.570
*Awareness* (α = 0.766, 3.75%)		
Media advertisement	0.700	0.641
Promotion through organisations	0.687	0.623
Product endorsement on label	0.653	0.604
Trust in the brand/product	0.648	0.492
Familiar to me	0.578	0.546
*Parent sensory* (α = 0.777, 2.88%)		
My texture preferences	0.764	0.658
My taste preferences	0.716	0.562
Smell	0.669	0.562
Appearance	0.650	0.592
*Convenience* (α = 0.844, 2.70%)		
Easy to prepare	0.876	0.793
Simple to cook	0.863	0.772
Preparation time	0.840	0.684
*Situation* (α = 0.775, 2.43%)		
Time of day	0.873	0.730
Time of meal	0.836	0.682
Activities of the day	0.686	0.589
*Culture* (C-α = 0.734, 2.29%)		
Harmony with my religious views	0.917	0.779
Rules and rituals relevant to my religion	0.904	0.786
Common in my culture	0.496	0.552
Fit within my social/cultural norms	0.480	0.557
*Professional advice* (α = 0.785, 2.09%)		
Advice from dietitian or nutritional professional	0.831	0.728
Advice from doctors, GPs or other medical professionals	0.768	0.705
Advice from exercise/fitness professionals	0.454	0.566
Accessibility (α = 0.703, 1.97%)		
Cost	0.785	0.667
Value for money	0.756	0.687
The availability in shops/supermarkets	0.665	0.551
The location of shops/supermarkets in relation to my work or home	0.537	0.519
*Weight control* (α = 0.664, 1.75%)		
Effect on my weight	0.671	0.535
Energy/calorie value	0.553	0.525
Effect on my child’s weight	0.506	0.557

**Table 3:** Intercorrelations (rs) between the Parent Carer Food Choice Factors (n = 737).

	Ethical Concerns	Convenience	Culture	Situation	Child Mood	Parent Sensory	Professional Advice	Weight Control	Nutritional Attributes	Accessibility	Awareness
Convenience	0.029										
Culture	0.382 ***	0.120 ***									
Situation	0.173 ***	0.156 ***	0.238 **								
Child mood	0.234 ***	0.260 ***	0.300 ***	0.374 ***							
Parent sensory	0.323 ***	0.203 ***	0.368 ***	0.229 ***	0.347 ***						
Professional advice	0.314 ***	0.114 **	0.285 ***	0.275 ***	0.374 ***	0.276 ***					
Weight control	0.417 ***	0.123 ***	0.333 ***	0.225 ***	0.395 ***	0.338 ***	0.415 ***				
Nutritional attributes	0.532 ***	−0.009	0.286 ***	0.396 ***	0.336 ***	0.258 ***	0.369 ***	0.494 ***			
Accessibility	0.209 ***	0.390 ***	0.232 ***	0.233 ***	0.324 ***	0.386 ***	0.221 ***	0.273 ***	0.139 ***		
Awareness	0.272 ***	0.253 ***	0.387 ***	0.308 ***	0.502 ***	0.385 ***	0.466 ***	0.375 ***	0.268 ***	0.307 ***	
Child preferences	0.064	0.315 ***	0.161 ***	0.369 ***	0.588 ***	0.239 ***	0.205 ***	0.222 ***	0.209 ***	0.263 ***	0.416 ***

*p* value; ** <0.01; *** <0.001.

## Data Availability

The raw data supporting the conclusions of this article will be made available by the authors on request.

## Supplementary Materials

The following supporting information can be downloaded at: https://www.mdpi.com/article/10.3390/nu17101735/s1; Document S1: PC-FCQ: The Parent and Carer Food Choice Questionnaire.
