# Supplementary material for: Development and Preliminary Validation of the PC-FCQ: The Parent and Carer Food Choice Questionnaire
Source: Nutrients. 2025 May 20;17(10):1735. doi: 10.3390/nu17101735 (PMC12114542; doi:10.3390/nu17101735)
Supplement: Supplementary file 1 [file nutrients-17-01735-s001.zip › nutrients-3601450-supplementary.pdf]

PC-FCQ : The Parent and Carer Food Choice Questionnaire

Please answer the following statements in relation to how much they influence your food choices. This could be a single meal or individual food or drink (beverage).

My food choices can be influenced by:	**Frequency**									
**Awareness**	**Never**									**Always**
Media advertisement	1	2	3	4	5	6	7	8	9	10
Promotion through organisations	1	2	3	4	5	6	7	8	9	10
Product endorsement on label	1	2	3	4	5	6	7	8	9	10
Trust in the brand / product	1	2	3	4	5	6	7	8	9	10
Familiar to me	1	2	3	4	5	6	7	8	9	10
**Culture**	**Never**									**Always**
Harmony with my religious views	1	2	3	4	5	6	7	8	9	10
Rules and rituals relevant to my religion	1	2	3	4	5	6	7	8	9	10
Common in my culture	1	2	3	4	5	6	7	8	9	10
Fit within my social / cultural norms	1	2	3	4	5	6	7	8	9	10
**Nutritional attributes**	**Never**									**Always**
Vitamin and mineral content	1	2	3	4	5	6	7	8	9	10
The amount of natural ingredients	1	2	3	4	5	6	7	8	9	10
The amount of artificial ingredients	1	2	3	4	5	6	7	8	9	10
Quality and freshness	1	2	3	4	5	6	7	8	9	10
Protein content	1	2	3	4	5	6	7	8	9	10
Effect on my child's health	1	2	3	4	5	6	7	8	9	10
Fibre (roughage) content	1	2	3	4	5	6	7	8	9	10
The amount of additives	1	2	3	4	5	6	7	8	9	10
Providing variety to my child / children	1	2	3	4	5	6	7	8	9	10
Fat content	1	2	3	4	5	6	7	8	9	10
**Child preferences**	**Never**									**Always**
My child's taste preferences	1	2	3	4	5	6	7	8	9	10
Acceptance by my child	1	2	3	4	5	6	7	8	9	10
Familiar to my child / children	1	2	3	4	5	6	7	8	9	10
Enjoyment by my child	1	2	3	4	5	6	7	8	9	10
My child's texture preference	1	2	3	4	5	6	7	8	9	10
Requested by my child	1	2	3	4	5	6	7	8	9	10
Previous food my child has eaten	1	2	3	4	5	6	7	8	9	10
Satisfies my child (fills them up)	1	2	3	4	5	6	7	8	9	10
**Child mood**	**Never**									**Always**
Helping my child relax	1	2	3	4	5	6	7	8	9	10
Keeping my child alert	1	2	3	4	5	6	7	8	9	10
Soothing my child	1	2	3	4	5	6	7	8	9	10
How tired they are	1	2	3	4	5	6	7	8	9	10
Helping to pacify my child	1	2	3	4	5	6	7	8	9	10
Rewarding my child	1	2	3	4	5	6	7	8	9	10
Easy for my child to eat	1	2	3	4	5	6	7	8	9	10

My food choices can be influenced by:	Frequency									
**Weight control**	**Never**									**Always**
Effect on my weight	1	2	3	4	5	6	7	8	9	10
Energy / calorie value	1	2	3	4	5	6	7	8	9	10
Effect on my child's weight	1	2	3	4	5	6	7	8	9	10
**Parent sensory**	**Never**									**Always**
My texture preferences	1	2	3	4	5	6	7	8	9	10
My taste preferences	1	2	3	4	5	6	7	8	9	10
Smell	1	2	3	4	5	6	7	8	9	10
Appearance	1	2	3	4	5	6	7	8	9	10
**Convenience**	**Never**									**Always**
Easy to prepare	1	2	3	4	5	6	7	8	9	10
Simple to cook	1	2	3	4	5	6	7	8	9	10
Preparation time	1	2	3	4	5	6	7	8	9	10
**Situation**	**Never**									**Always**
Time of day	1	2	3	4	5	6	7	8	9	10
Time of meal	1	2	3	4	5	6	7	8	9	10
Activities of the day	1	2	3	4	5	6	7	8	9	10
**Ethical concerns**	**Never**									**Always**
Pain inflicted on animals	1	2	3	4	5	6	7	8	9	10
Respecting animal rights	1	2	3	4	5	6	7	8	9	10
Being animal friendly	1	2	3	4	5	6	7	8	9	10
Freedom of movement of animals	1	2	3	4	5	6	7	8	9	10
The impact on the environment	1	2	3	4	5	6	7	8	9	10
Environmentally friendly production/manufacturing	1	2	3	4	5	6	7	8	9	10
CO2 emissions from production	1	2	3	4	5	6	7	8	9	10
Free range production	1	2	3	4	5	6	7	8	9	10
Environmentally friendly packaging	1	2	3	4	5	6	7	8	9	10
Disruption to the ecosystem	1	2	3	4	5	6	7	8	9	10
Produced in a country where human rights are not violated	1	2	3	4	5	6	7	8	9	10
**Accessibility**										10
Cost	1	2	3	4	5	6	7	8	9	10
Value for money	1	2	3	4	5	6	7	8	9	10
The availability in shops/supermarkets	1	2	3	4	5	6	7	8	9	10
The location of shops/supermarkets in relation to my work or home	1	2	3	4	5	6	7	8	9	10
**Professional advice**	**Never**									**Always**
Advice from dietitian or nutritional professional	1	2	3	4	5	6	7	8	9	10
Advice from doctors, GPs or other medical professionals	1	2	3	4	5	6	7	8	9	10
Advice from exercise / fitness professionals	1	2	3	4	5	6	7	8	9	10

Reference the use of this instrument to:
McKenna A, Thurecht RL, Swanepoel L, Blair G and Pelly FE. Development and Preliminary Validation of the PC-FCQ: The Parent and Carer Food Choice Questionnaire. *Nutrients*. 2025
